# Supplementary material for: Characterization of Salmonella spp. and E. coli Strains Isolated from Wild Carnivores in Janos Biosphere Reserve, Mexico
Source: Animals (Basel). 2022 Apr 20;12(9):1064. doi: 10.3390/ani12091064 (PMC9100909; doi:10.3390/ani12091064)
Supplement: Supplementary file 1 [file animals-12-01064-s001.zip › animals-1679472-supplementary.pdf]

Supplementary tables and figures

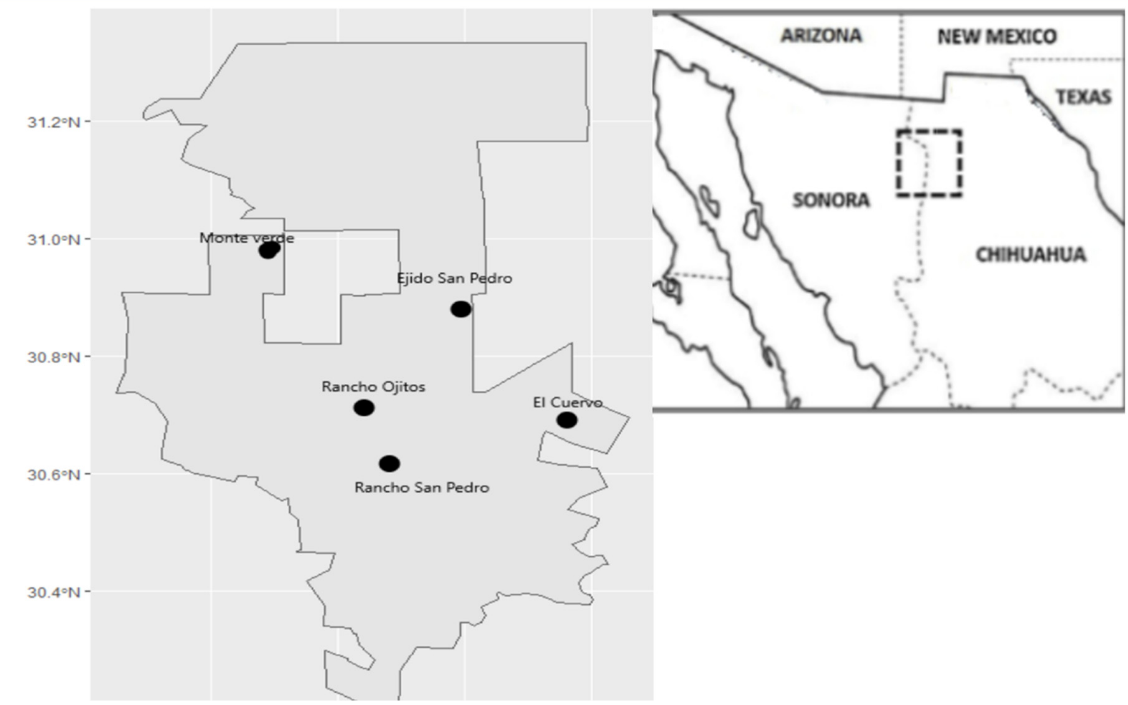

Supplementary Figure S1. Localization of the study area and sampling sites: a sampling point localization. Box with discontinuous borders represent study area (Janos Biosphere Reserve), black circles represent the five sampling locations.

Supplementary Table S1. Primer sequences and conditions for *Salmonella* and *E. coli* genetic characterization by PCR.

Name	Sequence (forward/reverse)	Cycle conditions	Amplicon	Ref.	Function
16ITS	5`-TGCGGCTGGATCACCTCCTT-3´/ 5´-TATAGCCCCATCGTGTAGTCAGAAC-3´	1 cycle 94 °C 5 min; 35 cycles 94 °C 1 min, 57 °C 1 min and 72 °C 2 min; 1 cycle 72 °C 5 min	312	[1]	component of the prokaryotic ribosome 30S subunit
*inv*A	5´-GTGAAATTATCGCCACGTTCGGGCAA-3´/ 5´-TCATCGCACCGTCAAAGGAACC-3´	1 cycle 94 °C 5 min; 35 cycles 94 °C 1 min, 57 °C 1 min and 72 °C 2 min; 1 cycle 72 °C 5 min	280	This study	component of the System secretion type III
*sop*E	5´-GTGACAAAAATAACTTTATCT-3´/ 5´-GGGAGTGTATTGTATATATTT-3´	1 cycle 94 °C 5 min; 35 cycles 94 °C 1 min, 43.5 °C 1 min and 72 °C 2 min; 1 cycle 72 °C 5 min	700	This study	Toxin associated with invasion
*cdt*B	5´-ATTTCTTTTGCCTGCGC-3´/ 5´-GATCAGAAGCGAGTTGTGG-3´	1 cycle 94 °C 5 min; 35 cycles 94 °C 1 min, 44 °C 1 min and 72 °C 2 min; 1 cycle 72 °C 5 min	700	This study	Subunit of the cytolethal distending toxin
*std*A	5´-ATGCGTAATAAAATAATACTTGCC-3´/ 5´-ATTTCAGGGTGTAGGTGACG-3´	1 cycle 94 °C 5 min; 35 cycles 94 °C 1 min, 50 °C 1 min and 72 °C 2 min; 1 cycle 72 °C 5 min	550	This study	Fimbrial protein
*lpf*A	5´-ATGGAGTTTTTAATGAAAAAGGTT-3´/ 5´-GGACAGGTTGAAGTCCACTT-3´	1 cycle 94 °C 5 min; 35 cycles 94 °C 1 min, 50 °C 1 min and 72 °C 2 min; 1 cycle 72 °C 5 min	310	This study	Fimbrial protein
*stf*A	5´-ATGAATACAGCAGTAAAAGCTG-3´/ 5´-ATCGTGAAGTTTACGGTGC-3´	1 cycle 94 °C 5 min; 35 cycles 94 °C 1 min, 50 °C 1 min and 72 °C 2 min; 1 cycle 72 °C 5 min	330	This study	Fimbrial protein
*bcf*A	5´-ATGAAAAAGCCTGTACTAGCATTAAT-	1 cycle 94 °C 5 min; 35 cycles 94 °C 1 min, 55 °C 1 min and	539	This study	Fimbrial protein

	3′/ 5′-GGAATAAACCATGCTAAATGTCG-3′	72 °C 2 min; 1 cycle 72 °C 5 min			
*sti*A	5′-ATGAAACTCTCCTTAAAAACACTCAC-3′/ 5′-CAGTTATATTGCAGATAGAATGTTGC-3′	1 cycle 94 °C 5 min; 35 cycles 94 °C 1 min, 55 °C 1 min and 72 °C 2 min; 1 cycle 72 °C 5 min	539	This study	Fimbrial protein
*stb*A	5′-ATGGCTGTTTCTGATAACACC-3′/ 5′-CAGCGACTGTCCCAGC-3′	1 cycle 94 °C 5 min; 35 cycles 94 °C 1 min, 55 °C 1 min and 72 °C 2 min; 1 cycle 72 °C 5 min	444	This study	Fimbrial protein
*ste*A	5′-ATGGGAATTGTCTCCGG-3′/ 5′-ACAGGTAAGAGATAGTGACGTTG-3′	1 cycle 94 °C 5 min; 35 cycles 94 °C 1 min, 55 °C 1 min and 72 °C 2 min; 1 cycle 72 °C 5 min	537	This study	Fimbrial protein
*fim*A	5′-ATGACCTCTACTATTGCGAGTCT-3′/ 5′-ATGATAAAGGTGGCGTCG-3′	1 cycle 94 °C 5 min; 35 cycles 94 °C 1 min, 55 °C 1 min and 72 °C 2 min; 1 cycle 72 °C 5 min	529	This study	Fimbrial protein
*sef*A	5′-ATGCGTAAATCAGCATCTGC-3′/ 5′-ATACTGCTGAACGTAGAAGGTCG-3′	1 cycle 94 °C 5 min; 35 cycles 94 °C 1 min, 55 °C 1 min and 72 °C 2 min; 1 cycle 72 °C 5 min	488	This study	Fimbrial protein
eae	3′-CGGGGACATTTTAGTCA-5′/ 5′-GATCAGAAGCGAGTTGTGG-3′	1 cycle 94 °C 5 min; 35 cycles 94 °C 1 min, 56 °C 1 min and 72 °C 2 min; 1 cycle 72 °C 5 min	600	This study	Intimin of E. coli
*esc*V	3′-GGCTCTCTTCTTCTTTATGGCTG-5′/ 5′-CCTTTTACAAACTTCATCGCC-3′	1 cycle 94 °C 5 min; 35 cycles 94 °C 1 min, 56 °C 1 min and 72 °C 2 min; 1 cycle 72 °C 5 min	534	[2]	component of the System Secretion type III
*bfp*B	3′-GATAAAACTGATACTGGGCAGC-5′/ 5′-AGTGACTGTTCGGGAAGCAC-3′	1 cycle 94 °C 5 min; 35 cycles 94 °C 1 min, 57 °C 1 min and 72 °C 2 min; 1 cycle 72 °C 5 min	826	[2]	Fimbrial protein

*stx*1	3′-GGCGTTCTTATGTAATGACTGC-5′/ 5′ -ATCCCACGGACTCTTCCATC-3′	1 cycle 94 °C 5 min; 35 cycles 94 °C 1 min, 57 °C 1 min and 72 °C 2 min; 1 cycle 72 °C 5 min	250	[2]	Subunit of Shiga toxin
*stx*2	3′-CGTTTTGACCATCTTCGTCTG-5′/ 5′-AGCGTAAGGCTTCTGCTGTG-3′	1 cycle 94 °C 5 min; 35 cycles 94 °C 1 min, 57 °C 1 min and 72 °C 2 min; 1 cycle 72 °C 5 min	325	[2]	Subunit of Shiga toxin
st	3′-TAGAGACCGGTATTACAGAAATCTGA-5′/ 5′-TCATCCCGAATTCTGTTATATATGTC-3′	1 cycle 94 °C 5 min; 35 cycles 94 °C 1 min, 60 °C 1 min and 72 °C 2 min; 1 cycle 72 °C 5 min	282	This study	Thermostable toxin
lt	3′-GGGTTGGCAATTTTTATTTCTGTA-5′/ 5′-ATTACAACAAAGTTCACAGCAGTA-3′	1 cycle 94 °C 5 min; 35 cycles 94 °C 1 min, 60 °C 1 min and 72 °C 2 min; 1 cycle 72 °C 5 min	183	This study	Thermolabile toxin

1. Chiu, T.H.; Chen, T.R.; Hwang, W.Z.; Tsen, H.Y. Sequencing of an internal transcribed spacer region of 16S-23S rRNA gene and designing of PCR primers for the detection of *Salmonella* spp. in food. *Int. J. Food Microbiol.* **2005**, *97*, 259–265.
2. Müller, D.; Hagedorn, P.; Brast, S.; Heusipp, G.; Bielaszewska, M.; Friedrich, A.W.; Karch, H.; Schmidt, M.A. Rapid identification and differentiation of clinical isolates of enteropathogenic *Escherichia coli* (EPEC), atypical EPEC, and Shiga toxin-producing *Escherichia coli* by a one-step multiplex PCR method. *J. Clin. Microbiol.* **2006**, *44*, 2626–2629.

Supplementary Table S2. Characteristics of *E. coli* strains isolated from carnivores.

Specie	Strain name	Serotype		Virulence genes	Phylogroup
		**O**	**H**		
Vulpes macrotis	20-BVul4	O153	H21	*escV-eae*	Unknown
Vulpes macrotis	20-BVul5	O153	H21	*eae*	Unknown
Vulpes macrotis	20-BVul6	O153	H21	*escV-eae*	Unknown
Vulpes macrotis	20-BVul7	O153	H21	*escV-eae*	Unknown
Vulpes macrotis	20-BVul8	O153	H21	*escV-eae*	Unknown
Vulpes macrotis	20-BVul12	O153	H21	*escV-eae*	E
Vulpes macrotis	20-BVul25	O153	H21	*escV-eae*	Unknown
Vulpes macrotis	20-BVul28	O153	H21	*Eae*	Unknown
Vulpes macrotis	20-Bvul30	O175	H16	*escV*	Unknown
Vulpes macrotis	39-BrVul28	O139	H21	*escv*	Unknown
Vulpes macrotis	39-BrVul18	O139	H21	*eae*	Unknown
Vulpes macrotis	39-BrVul20	O139	H21	*eae*	Unknown
Vulpes macrotis	39-BrVul31	O139	H21	*escV*	Unknown
Vulpes macrotis	39-BrVul5	O153	H7	*escV*	Unknown
Vulpes macrotis	39-BrVul7	O153	H7	*escV*	Unknown
Vulpes macrotis	39-BrVul8	O153	H7	*escV*	Unknown
Vulpes macrotis	39-BrVul23	O153	H7	*escv*	Unknown
Vulpes macrotis	39-BVul23	O153	H7	*eae*	Unknown
Vulpes macrotis	39-BVul24	O153	H7	*eae*	Unknown
Vulpes macrotis	39-BrVul25	O153	H7	*escV*	Unknown
Vulpes macrotis	39-BrVul26	O153	H7	*escV*	D
Vulpes macrotis	39-BrVul29	O153	H7	*escV*	E
Vulpes macrotis	39-BrVul27	O139	H?	*escV*	Unknown
Canis latrans	26-BCoy3	O75	H38	*stx1-stx2*	Unknown
Canis latrans	26-BCoy19	O75	H38	*stx1-stx2*	Unknown
Canis latrans	26-BCoy6	O17	H18	*stx1-stx2*	Unknown
Canis latrans	35-BCoy26	O21	H21	*stx2*	Unknown
Canis latrans	35-BCoy27	O21	H21	*stx2*	Unknown
Canis latrans	35-BCoy35	O21	H21	*stx2*	Unknown
Canis latrans	35-BCoy36	O21	H21	*stx2*	Unknown
Canis latrans	Coy 3-19	O110	H28	*stx1-stx2*	Unknown
Canis latrans	Coy 3-31	O110	H28	*stx1-stx2*	Unknown
Canis latrans	35-BCoy5	O ?	H11	*stx2*	Unknown
Canis latrans	35-BCoy9	O ?	H11	*stx2*	Unknown
Canis latrans	35-BCoy14	O ?	H11	*stx2*	Unknown
Canis latrans	35-BCoy16	O ?	H11	*stx2*	D
Canis latrans	35-BCoy17	O ?	H11	*stx2*	Unknown

Canis latrans	35-BCoy21	O ?	H11	*stx2*	Unknown
Canis latrans	35-BCoy23	O ?	H11	*stx2*	Unknown
Canis latrans	35-BCoy24	O ?	H11	*stx2*	D
Canis latrans	35-BCoy28	O ?	H11	*stx2*	Unknown
Canis latrans	35-BCoy30	O ?	H11	*stx2*	Unknown
Canis latrans	35-BCoy31	O ?	H11	*stx2*	D
Canis latrans	35-BCoy32	O ?	H11	*stx2*	Unknown
Canis latrans	35-BCoy33	O ?	H11	*stx2*	Unknown
Mephitis macroura	Meph 15-2	O63	H6	*escv*	B2
Mephitis macroura	Meph 15-21	O63	H6	*escv*	B2
Mephitis macroura	Meph 15-22	O63	H6	*escv*	B2
Mephitis macroura	Meph 15-23	O63	H6	*escv*	B2
Mephitis macroura	Meph 15-8	O63	H6	*escv*	B2
Mephitis macroura	Meph 15-27	O63	H6	*escv*	B2
Mephitis macroura	Meph 15-28	O63	H6	*escV-eae*	B2
Mephitis macroura	Meph 15-5	O96	H49	*escv*	B2
Mephitis macroura	Meph 15-6	O96	H49	*escv*	B2
Mephitis macroura	Meph 15-24	O96	H49	*escv*	B2
Mephitis macroura	Meph 15-12	O96	H49	*escv*	D
Mephitis macroura	Meph 18-7	O93	H?	*stx1*	A
Mephitis macroura	Meph 18-8	O93	H?	*stx1*	A
Mephitis macroura	Meph 18-17	O93	H?	*stx1*	A
Mephitis macroura	Meph 15-12	nd	nd	*escv*	B2
Mephitis macroura	Meph 15-1	nd	nd	*escV*	B2
